# Micro- and nanoplastics influences in Parkinson’s disease: lessons from human stem cell models

**DOI:** 10.3389/ftox.2026.1723092

**Published:** 2026-03-26

**Authors:** Kamilah Na’imah Muhammad, Tracey Woodlief, Xian Wu

**Affiliations:** Department of Pharmacology and Toxicology, Brody School of Medicine, East Carolina University, Greenville, NC, United States

**Keywords:** dopaminergic neuron, microglia, microplastic, nanoplastic, Parkinson’s disease, stem cell

## Abstract

Neuroinflammatory contributions play a critical role in Parkinson’s disease onset and progression. Key drivers of neuroinflammation include glial cell reactivity, cytokine signaling, protein aggregation, and mitochondrial dysfunction. Although animal models have been extensively used to investigate the mechanisms, their translational relevance is limited because neuroinflammation in humans is typically chronic, heterogeneous, and sustained over years, whereas in rodents is often acute, transient, and resolves within days to weeks. This paper highlights the utility of human stem cell–derived models in studying Parkinson’s disease by recapitulating patient-specific genetic mutations, neuroinflammatory microglia–neuron interactions, α-synuclein aggregation, and dopaminergic dysfunction, thereby enabling mechanistic studies in the human-relevant models. In addition, we examine how micro- and nanoplastics may exacerbate neuroinflammation in PD. This review concludes by highlighting how human-relevant stem cell-based approaches advance mechanistic understanding of Parkinson’s disease.

## 1 Introduction

Parkinson’s Disease (PD) is a progressive neurodegenerative disorder characterized by motor dysfunctions, including tremor, rigidity, and bradykinesia, as well as non-motor symptoms (Sveinbjornsdottir, 2016). The loss of dopaminergic neurons in the substantia nigra and the accumulation of Lewy bodies are hallmark pathological features of the disease. In addition to these mechanisms, neuroinflammation has emerged as a critical player in the pathogenesis of PD (Wang et al., 2025).

This review explores the role of stem cell models in PD and highlights the extent to which micro- and nanoplastics (MNPs) contribute to the pathology of the disease. *In vivo* studies demonstrate that exposure to MNPs correlates with neurodegenerative risk (Ma et al., 2026; Bayattork et al., 2026; Hammond et al., 2021). Stem cell models offer key advantages when examining the causes and contributors of PD at the cellular and organelle level.

## 2 Neuroinflammation in Parkinson’s disease

Building on the established role in PD pathogenesis, neuroinflammation involves an integrated response mediated by glial activation, immune signaling, and cellular stress pathways. In Parkinson’s disease, neuroinflammatory processes are driven by mechanisms of excitotoxicity, protein aggregation, mitochondrial dysfunction (Iovino, Tremblay, and Civiero, 2020; Mahadevan et al., 2021; Calabresi et al., 2023; Funayama et al., 2023).

### 2.1 Glial cell activation

**FIGURE 1:**
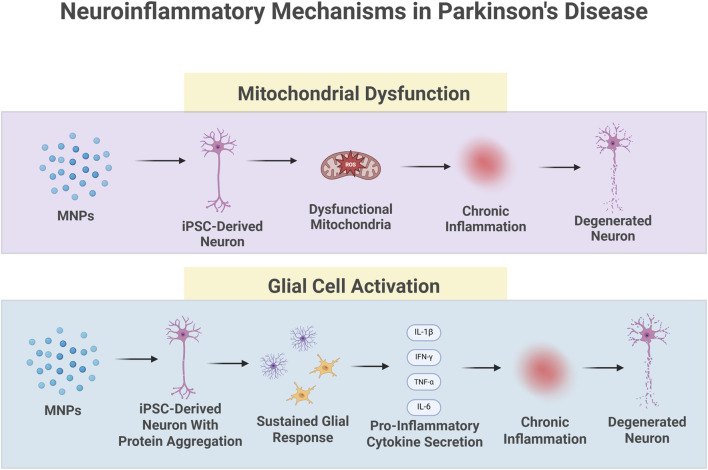
Contributing factors to neuroinflammation. Toxicant exposure to the cell can lead to dysfunction of the mitochondria, which leads to inflammation and neural degeneration. Glial hyperactivity contributes to neural degeneration through pro-inflammatory cytokines. Created in BioRender. Wu, X. (2026) Contributing factors to neuroinflammation, https://BioRender.com/wpt5vxu.

Microglia, the brain’s resident immune cells, are central to this process. They are activated by Toll-like receptors (TLRs), which detect a pathogen-associated molecular pattern (PAMP) or damage-associated molecular pattern (DAMP) (Yang et al., 2020). Once activated, TLRs signal microglia, recruit T-cells, and stimulate cytokine release. Dysfunction in TLR signaling may impair immune detection and resolution, sustaining chronic neuroinflammation. While acute microglia activation is beneficial, chronic activation promotes oxidative stress and excitotoxicity, accelerating dopaminergic neuron loss (Figure 1) (Iovino, Tremblay, and Civiero, 2020). Microglia derived from human stem cells are increasingly used to model microglial activation, enabling direct investigation of how environmental toxicants trigger chronic neuroinflammation and exacerbate dopaminergic neuron vulnerability in PD (Abud et al., 2017). Astrocytes also contribute by detecting pathogens and modulating cytokine release, further amplifying the inflammatory environment (Iovino, Tremblay, and Civiero, 2020).

### 2.2 Cytokine signaling

Furthermore, the glia-driven inflammation is closely linked to increased cytokine signaling in PD. Individuals with PD exhibit elevated levels of TNF-α, IL-1β, and IFN-γ, in cerebrospinal fluid (Zimmermann and Brockmann, 2022; Blum-Degen et al., 1995; Mogi et al., 1994). Immune mechanisms such as antibody-dependent cell-mediated cytotoxicity (ADCC) and upregulation of Leucine-Rich Repeat Kinase 2 (LRRK2), key immune regulator, have also been implicated (Hunot and Hirsch, 2003). Human iPSC-derived dopaminergic neuron and immune co-culture models were used to investigate how elevated cytokines such as TNF-α and IL-1β, as well as LRRK2-associated immune signaling, sensitize neurons to environmental toxicants, providing a human-relevant platform to dissect whether immune activation acts as a driver or consequence of PD pathology (Stoberl et al., 2023). Although fluctuations in cytokine levels are correlated with PD, it is still unknown if they are a cause or symptom of the disease.

### 2.3 α -synuclein aggregation

Beyond soluble inflammatory mediators, alpha-synuclein which is vital for neuronal cell signaling becomes pathogenic when it aggregates into Lewy bodies, which contribute to neuroinflammation and ultimately neuronal cell death (Grozdanov et al., 2019). These aggregates vary in their immune effects depending on size, conformation, and surface proteins (Grozdanov et al., 2019). Alpha-synuclein can adopt other forms sch as oligomers, fibrils, and protofibrils, which can also contribute to disease pathologies (Calabresi et al., 2023). Increased levels of autoantibodies against alpha-synuclein have also been found in PD patients, which can partially account for its role in neuroinflammation (Tansey et al., 2022). However, similar antibody elevations have also been found to be increased in response to dopamine, which suggests that it is not particularly selective to protein aggregates in nature. In addition, extracellular vesicles have been found to have a facilitatory role in the release and spread of alpha-synuclein (Grozdanov et al., 2019), potentially acting as transport vehicles that propagate pathology throughout the brain. Alpha-synuclein aggregation can be modeled in human iPSC lines possessing SNCA mutations, which allows for the study of molecular implications of protein misfolding and comparison to healthy cell lines (Iannielli et al., 2022).

### 2.4 Mitochondrial dysfunction

Bioenergetic dysfunction is increasingly recognized as a contributing factor to PD. Protein alterations, including α-synuclein aggregation, can impair mitochondrial efficiency, leading to oxidative stress (Li et al., 2023). Accumulation of α-synuclein can impair complex I activity and disrupt mitochondrial respiration and cellular bioenergetics (Devi et al., 2008). Several proteins, such as PARK7, PARK2, and PINK1, are associated with both PD and mitochondrial dysfunction (Mahadevan et al., 2021), highlighting a mechanistic intersection between PD pathology and mitochondrial disruption. Human pluripotent stem cell-derived neurons carrying PARK2 or PINK1 dysfunction have been widely used to model impaired mitophagy and oxidative stress responses following exposure to environmental neurotoxins, providing a human-relevant platform to investigate how mitochondrial vulnerability contributes to PD progression (Avazzadeh et al., 2021). Mutations in PINK1 and PARK2 have been strongly linked to mitochondrial quality control pathways that regulate mitochondrial dynamics and mitophagy in PD. Experimental studies demonstrate that PARK2, PINK1, and α-synuclein converge on mitochondrial stress response pathways, influencing mitochondrial dynamics and cellular bioenergetics under conditions of mitochondrial damage (Norris et al., 2015). These findings highlight the importance of PINK1/PARK2 signaling in maintaining mitochondrial integrity and provide mechanistic insight into how disruptions in these pathways contribute to PD pathology. Additionally, a clinical study has found that dopaminergic neurons derived from the cells of PD patients demonstrate differences in basal respiration and reserve capacity compared to controls (Barnhoorn et al., 2024).

These mitochondrial abnormalities are particularly evident in microglial cells. In microglia, inflammatory responses are closely tied to shifts in mitochondrial function, including altered ROS production and membrane potential (Ye et al., 2017). Excessive α-synuclein aggregation in the brain activates the microglial autophagy process, which is associated with a “defective turnover” of mitochondria (Ye et al., 2017). As a result, damaged mitochondria accumulate within microglia, further amplifying ROS production and sustaining the neuroinflammatory response (Ye et al., 2017). In sum, misfolded proteins such as α-synuclein impair energy metabolism, which contributes to immune activation and neurodegeneration (Figure 1).

Together, these interconnected mechanisms highlight the complexity of neuroinflammation in PD and underscore the need for human-relevant models capable of modeling neuroinflammation in PD with environment interactions, motivating the use of human stem cell–based systems to investigate these interacting mechanisms.

## 3 Human stem cells as models for Parkinson’s disease

### 3.1 2D models of Parkinson’s disease

Two-dimensional (2D) culture systems provide a simplified and highly controllable platform that allows precise manipulation of neural cell populations, including cellular composition, genetic background, and environmental cues (Centeno, Cimarosti, and Bithell, 2018). Unlike conventional monoculture approaches, 2D human stem cell co-culture systems enable direct cell–cell interactions between neurons and microglial populations while preserving accessibility for high-throughput imaging and analyses (Liu et al., 2022; Wevers et al., 2016).

The use of stem cell technology to develop disease models has greatly advanced scientific research and holds significant promise for future therapeutic interventions. Distinct types of stem cells including embryonic stem cells (ESCs) and human induced pluripotent stem cells (hiPSCs) are employed in PD research due to the ability to proliferate and differentiate particularly in generating dopaminergic cells for modeling (Chen, and Li, 2015). A key advantage of hiPSCs is their capacity to capture diverse genetic backgrounds, as they can be directly generated from human patients (Smits et al., 2019). The hiPSC-based models also allow in depth high throughput screening of drug candidates and their mechanisms without relying on patient tissue samples (Kim et al., 2025).

Recently, 2D cell culture models integrating microglia into neural culture have been conducted to study the inflammatory effect that microglia have on dopaminergic neurons. Though there are currently a limited number of studies exploring this issue, experiments thus far have demonstrated a plausible pro-inflammatory interaction. A cell culture study found that integrating microglia containing mutations such as LRRK2 G2019s have increased locomotive phagocytotic abilities as opposed to controls (Kim et al., 2019). Other potential microglial contributions include maladaptive processing of misfolded neural proteins and impairment of synaptic pruning (Kim et al., 2019). A recent hypothesis on microglial contributions to neuroinflammation and PD are based on transcriptome analysis (Kim et al., 2025). Thus, co-culture studies are necessary to confirm the extent of crosstalk between microglia and dopaminergic neurons and their contribution to PD.

In all, 2D stem cell models of PD have contributed much to the degenerative neuroscientific field. The ability to study specific features of the disease at a cellular level has provided much insight. In terms of future directions, there are still many genes involved in PD that require study such as UCHL-1, Parkin, DJ-1, PAPK3, and PAPK13 (Zhang et al., 2017). Future research will allow for in-depth study of these mechanisms. In clinical practice, there is still uncertainty regarding whether these models can result in effective transplantation treatment for patients. While many new mechanisms for transplantation are currently in development, there is still potential for these interventions to cause adverse immune responses in patients (Zhang et al., 2017).

**FIGURE 2:**
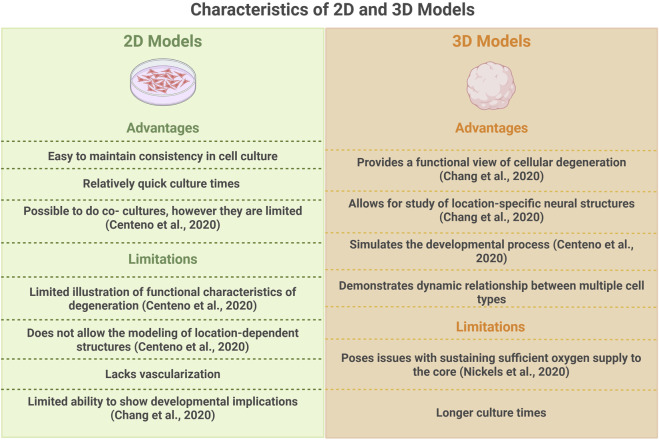
A Comparison of 2D and 3D stem cell models. While 2D iPSC models are a convenient method to study molecular characteristics of a cell type, 3D iPSC models allow for the investigation of functional interactions between cell types. Created in BioRender. Wu, X. (2026) https://BioRender.com/wpt5vxu.

Although valuable information can be obtained from 2D models, these methods cannot fully replicate the complexities of the nervous system, which limits their efficacy (Figure 2). Traditional 2D cultures are limited by lacking functional interactions between glial cells and neurons (Smits et al., 2019).

### 3.2 3D models of Parkinson’s disease

3D organoid chimeras can recapitulate functional interactions between neural and glial cells in neurodegenerative models, allowing for a multifaceted approach to studying contributing factors to degeneration. Importantly, these multicellular organoid systems also enable investigation of neuroinflammatory mechanisms by capturing dynamic interactions among neurons, astrocytes, and microglia cells (Soler et al., 2022). Additionally, when midbrain organoids are cultured from expandable floor plate neural progenitor cells, they can be directed to express phenotypes that are prevalent in patients diagnosed with PD (Smits et al., 2019). This allows for the study of transcriptomic alterations that may contribute to disease. Human midbrain organoids express key midbrain-specific and dopaminergic neuron markers such as FOXA2, LMX1A, tyrosine hydroxylase (TH), and dopamine transporter (DAT), and exhibit neuronal differentiation and functional features representative of midbrain development (Nickels et al., 2020; Monzel et al., 2017). In a comprehensive study by Kim et al. (2019), the G2019S mutation in LRRK2 was modeled to make a 3D model of PD in midbrain organoids (Kim et al., 2019). This mutation resulted in cells being unable to clear excess protein accumulations, leading to α-synuclein aggregation. Additionally, LRRK2 stem cell derived organoids expressed TH, AADC, and DAT in significantly lower amounts than controls, which suggests decreased dopaminergic activity. Gene set enrichment analysis (GSEA) showed that LRRK2 organoids showed genetic enhancements that were similar to those in PD patient brain tissue (Kim et al., 2019). This study further revealed that dysregulating the TNX1P gene contributed to α-synuclein aggregation. These cells were also more vulnerable to damage when exposed to MPTP, which points to the possibility of decreased resilience against environmental toxins (Kim et al., 2019).

The consideration of 3D models into therapeutic approaches demonstrates their relevance to the field of neurodegenerative research. One study posits that human midbrain organoids can be used as donor grafts to treat PD (Zheng et al., 2023). In this study, organoids that matured to express dopaminergic neurons were transplanted into mice with 6-OHDA lesions. It was found that after transplantation, these animals showed improvements in motor function. Additionally, these midbrain organoids were able to develop projections, enabling integration into the host nervous system (Nickels et al., 2020). Thus, organoids show much promise in their ability to model functional interactions, contributing to disease pathology (Heydari et al., 2021). In terms of therapeutics, the idea of using organoids possessing mature dopaminergic neurons as donor grafts to restore motor function is also an emerging area of research (Fu et al., 2024). However, there is still much research that must be done on 3D organoid models to improve their ability to mimic the human brain environment (Figure 2).

## 4 Micro- and nanoplastics and neuroinflammation

Microplastics and nanoplastics are prevalent sources of pollution that are implicated in neuroinflammation. Human consumption, environmental contamination, and retail packaging are common sources of human MNP exposure (Cox et al., 2019). In the United States, common sources of MNPs include polycarbonate, polyurethane, polystyrene, polyethylene terephthalate, polyvinyl chloride, polyethylene, and polypropylene (Kannan and Vimalkumar 2021). Plastic waste can also stem from cotton, polyester, polyolefin, PTFE, PE, PP, nylon, and synthetic polymers (Kannan and Vimalkumar 2021). Notably high concentrations of MNP contamination are common in food items such as seafood, honey, alcohol, salt, sugar, and drinking water (Cox et al., 2019). In terms of their ability to infiltrate the human body, MNPs are quite capable, with annual human consumption from food and drink ranging from 39,000 to 52,000 particles and annual inhalation ranging between 35,000 and 62,000 (Cox et al., 2019).

The ability of MNPs to be ingested, translocated, and accumulated in the human body is primarily responsible for their potential contribution to neurodegenerative pathologies. Microplastics smaller than 130 μm can translocate into human tissue and thus trigger a pro-inflammatory immune response (Cox et al., 2019). A key factor of MNP ingestion that accounts for its pro-inflammatory abilities is that these particles cannot be readily broken down by chemical or biological means (Kannan and Vimalkumar 2021). The resistance of these particles to the body’s typical means of waste elimination mediates a pro-inflammatory response in the body’s attempt to degrade the foreign material.

In a comprehensive study by Nihart et al. (2025) on human brain samples derived from the frontal cortex found that brains had larger concentrations of MNPs than in the liver and kidney (Nihart et al., 2025), suggesting that more plastic is being accumulated in the brain as opposed to being excreted from the body. Research has shown that patients can potentially have higher MNP concentrations in their brain than healthy individuals (Nihart et al., 2025). Additionally, an overall increase in MNP concentrations in the brain over the span of 8 years suggests that MNP-related pathologies may increase in prevalence (Nihart et al., 2025).

A mechanistic study on MNPs in PD suggests that chemical interactions between α-synuclein and anionic nanoplastic could be a key factor in disease pathology (Liu et al., 2023). It is posited that the negatively charged nanoplastics interact with the non-amyloid component (NAC) of α-synuclein due to this area having an abundance of positively charged lysine particles (Liu et al., 2023). The ionic bonding that occurs between nanoplastic and the NAC potentially facilitates leakage in the endothelial tissue, breaches in the blood-brain barrier, and domain rearrangements that encourage protein fibrils prone to bind to nanoplastics (Liu et al., 2023). Nanoplastics can also directly impact neurons through clathrin-dependent internalization, resulting in lysosomal impairment (Liu et al., 2023). When lysosomes become impaired, the cell’s ability to effectively clear α-synuclein is reduced, promoting aggregation.

MNP studies using stem cell models demonstrate other possible mechanisms for impairment. Polystyrene MNPs penetrating the cellular membrane after prolonged exposure in hiPSC-derived cerebral organoids (Tao et al., 2024; Hua et al., 2022). As a consequence of nanoplastic exposure, mitochondrial dysfunction with key genes such as TP53 PARP1, METTL4 being downregulated (Tao et al., 2024). Deficits in differentiated neurons and decreased organoid size are also associated with a decline in neural activity (Tao et al., 2024). Conversely, short-term microplastic exposure appears to encourage cell proliferation and gene expression (Hua et al., 2022). These key findings from hiPSC studies point out potential mechanisms of cellular death and neurodegeneration due to MNP exposure and require further investigation on whether MNPs alter inflammatory cell viability in ways that promote neurodegeneration.

Thus, MNPs are a widespread contaminant that can contribute to PD through various mechanisms (Liu et al., 2023; Tao et al., 2024; Hua et al., 2022). Through their ability to bind to α-synuclein, they can form tight interactions that impair the body’s endothelial immune barriers, resulting in migration to and throughout the brain. These interactions can result in an increase in α-synuclein formation, which would intensify the brain’s immune response. Studies on brain samples support the idea that dysfunction of the blood brain barrier and clearance mechanisms facilitate MNP accumulation in the brain (Wang et al., 2026; Gecegelen, Ucdal, and Dogu, 2025). This same mechanism could possibly result in the accumulation of α-synuclein in the brain, resulting in an aggressive immune response that causes neuroinflammation and cell death. Stem cell models of MNP exposure reinforce this hypothesis, showing genetic dysregulation following MNP infiltration into organoid tissue. Current findings on MNP-related dysfunction are largely correlative, and the underlying mechanisms remain hypothetical and incompletely defined, underscoring the need for advanced stem cell–derived systems to elucidate causal links between MNP exposure and Parkinson’s disease pathology.

## 5 Challenges and future perspectives

*In vitro* stem cell models still face challenges in capturing the complexity of PD despite current significant progress. A major disadvantage is the difficulty of modeling neurodegeneration and neuroinflammation simultaneously, as current 2D models only partially recapitulate cytokine signaling, α-synuclein aggregation, and metabolic or genomic alterations. Although 3D organoid models enable structural and functional analyses, the lack of vascularization results in necrotic cores and insufficient oxygen, limiting physiological relevance (Nickels et al., 2020). Establishing a direct causal link between MNPs exposure and PD remains challenging, as contaminant-induced neuroinflammatory and mitochondrial stress responses may reflect general neurotoxicity rather than PD-specific pathology. Moreover, organoid models typically represent discrete brain regions, complicating the study of region-specific vulnerability such as striatal degeneration.

Recent advances in stem cell technologies provide promising avenues for future research. Stem cell models focused on neurodegenerative pathologies such as Alzheimer’s disease and amyotrophic lateral sclerosis show great potential for an iPSC model of PD (Barak et al., 2022; Giacomelli et al., 2022). However, current iPSC studies of MNP exposure model acute rather than chronic exposure. Optimizing production of midbrain organoids that lack necrotic cores would provide a key advantage in studying PD. Additionally, the synthesis of new methodologies to generate vascularized brain organoids would fundamentally improve the efficacy of 3D organoid models. Organoid models consisting of vasculature and simulating multiple brain regions would be ideal for studying the effects of chronic MNP exposure. Animal models are likely a vital accessory to 3D organoid models in the onset of toxicology research (Li et al., 2022). It is most efficient to first determine if toxicants target location-specific structures to the brain before resources are allocated to generating 3D organoids.

## 6 Conclusion

Human stem cell models provide a robust platform for investigating mechanisms in Parkinson’s disease. Among various stem cell types hiPSCs are particularly valuable due to their ability to differentiate into multiple neural and glial cell types, closely recapitulating disease-relevant cellular contexts (Centeno, Cimarosti, and Bithell, 2018).

Neuroinflammation is a key contributor to PD pathology, driven by mechanisms such as microglial activation, increased cytokine levels, and α-synuclein aggregation (Iovino, Tremblay, and Civiero, 2020; Mahadevan et al., 2021; Calabresi et al., 2023; Funayama et al., 2023). MNPs may exacerbate neuroinflammatory responses by crossing the blood–brain barrier, interacting with brain organelles, promoting protein aggregation, activating glial cells, and increasing pro-inflammatory cytokine production. These contaminants represent important targets for understanding PD progression and identifying preventive strategies. Stem cell–based neurodegenerative models allow investigation of Parkinson’s disease cellular mechanisms while integrating environmental MNPs toxicology to provide a more realistic representation of the multifactorial nature of disease pathology.
